# Preputial and scrotal cutaneous mast cell tumors in dogs show no evidence of inherently higher biologic malignancy

**DOI:** 10.3389/fvets.2025.1672099

**Published:** 2025-10-29

**Authors:** Žiga Žagar, Axel Wehrend, Jarno M. Schmidt, Sandra Kuehnel-Lawatsch, Tabea Buehler, Wolf von Bomhard, Martin Kessler

**Affiliations:** ^1^IVC Evidensia Small Animal Clinic Hofheim, Hofheim am Taunus, Germany; ^2^Clinic of Obstetrics, Gynaecology and Andrology of Large and Small Animals, Justus-Liebig-University Giessen, Giessen, Germany; ^3^Tierpathologie München, Antech Lab Germany GmbH, Munich, Germany

**Keywords:** canine, genital, MCT, outcome, prognosis, Ki-67, KIT

## Abstract

Canine genital mast cell tumors (MCTs) have been associated with a poorer prognosis; however, no larger study has focused exclusively on MCTs in this region. This study aimed to retrospectively describe the clinicopathologic aspects and outcomes of dogs with cutaneous preputial and scrotal MCTs and compare the findings to historical data from cutaneous MCTs from other locations. Medical records from 2002 to 2024 from a single institution were reviewed and 91 dogs (35 preputial, 56 scrotal) treated surgically with or without adjuvant therapy and a minimum follow-up of 6 months were included. Tumors were graded according to Patnaik (preputial: 63% grade I, 31% grade II, 6% grade III; scrotal: 41% grade I, 38% grade II, 21% grade III) and Kiupel (preputial: 91% low-grade, 9% high-grade; scrotal: 79% low-grade, 21% high-grade). Histological evaluation of superficial inguinal lymph nodes was performed in 55% of cases (50 dogs); of these, 16% (8/50) showed HN3 metastases. The overall median survival time was not reached and the 1-, 3- and 5-year survival rates were 85%, 67%, and 60%, respectively. On multivariable analysis, Kiupel high-grade and tumor diameter of at least 2 cm were associated with a shorter overall survival time, while HN3 lymph node metastases, aberrant KIT staining pattern, and Ki-67 index >23 were not. This data does not provide evidence of an inherently high biologic aggressiveness of preputial and scrotal MCTs. In the absence of other negative prognostic factors, dogs with preputial and scrotal MCTs have a favorable prognosis.

## 1 Introduction

The biologic behavior of canine cutaneous mast cell tumors (MCTs) varies greatly from indolent masses to highly malignant, rapidly growing tumors with high recurrence and metastatic rates. While several factors have been associated with the biological behavior of cutaneous MCTs and disease outcomes, no single indicator is fully predictive or prognostic (1–3).

Several anatomic locations, such as the muzzle, oral mucosa, mucocutaneous junctions, subungual area, pinna, inguinal, perineal, and genital regions have been proposed to harbor biologically more aggressive MCTs compared to other cutaneous locations (1, 4–10). The existence of different subpopulations of mast cells in various anatomic locations has long been known (11, 12), which could explain the differences in biologic behavior of MCTs in distinct anatomic regions. Even though not specifically supported by current evidence for genital locations, hypothetically, differences in lymphatic drainage patterns across various anatomical regions could influence the rate or pattern of nodal metastasis.

To the best of the authors’ knowledge, no larger study to date has focused exclusively on cutaneous MCTs in the genital area of male dogs, and the perception of worse prognosis for these anatomic regions has not been conclusively demonstrated and warrants further investigation. A shorter disease-free interval was reported in 12 dogs with preputial and scrotal MCTs compared to MCTs from other anatomic locations (5). Two other studies found a higher likelihood of high-grade tumors in perigenital, scrotal and inguinal regions (6, 7). On the other hand, no difference in mean survival time was found in 68 dogs with inguinal and perineal cutaneous MCTs when compared to historical data of MCTs from other anatomic locations (13).

Especially on the prepuce, wide surgical resections and functional reconstructions are anatomically challenging, and, as long as recommendations regarding the extent of surgery are based on anecdotal data, dogs might be unjustifiably subjected to invasive therapeutic procedures.

Due to the lack of data for evidence-based treatment decisions, the clinical and potentially prognostic impact of MCTs in these areas is of great relevance. The primary aim of this retrospective, single-center, observational study was to describe the clinicopathologic aspects in dogs with cutaneous MCTs originating from the preputial (pMCT) and scrotal (sMCT) regions and evaluate their outcomes. We compared the findings to historical data on MCTs from other cutaneous locations. Our secondary aim was to assess the influence of potential prognostic variables on overall survival time.

## 2 Materials and methods

### 2.1 Data collection

The medical records of a large small animal referral hospital were retrospectively reviewed to identify dogs with cutaneous preputial and scrotal MCTs presented between January 1, 2002 and April 10, 2024. The database was searched for “mastzelltumor” (mast cell tumor) or “mct” and “skrot” (scrotal) or “scrot” or “präput” (preputial). To be included in the study, dogs had to have undergone a surgical resection of the MCT with or without adjuvant therapy. For inclusion, a histopathologic report including Patnaik (14) and Kiupel (15) grading had to be available for review, and grading was performed according to internationally accepted criteria for canine cutaneous mast cell tumors (16). Dogs with subcutaneous or mucosal MCTs were excluded. The minimum follow-up was 6 months.

The scrotum was defined as the pouch of skin between the thighs from its most distal part to the cutaneous border adjoining the trunk. The preputium was defined as the fold of haired skin (external lamina) covering the penis from its tip to the border adjoining the trunk (17). Tumors defined as periscrotal or peripreputial by the attending clinician were excluded.

Data obtained from the medical records included age, breed, neuter status, size, number, clinical appearance and histological grade of the tumor, presence of pruritus, mitotic count per 10 high-power fields (2.37 mm^2^), KIT and Ki-67 status, extent of staging performed and its results, completeness of the surgical resection, information on relapse, time of tumor progression (if available) and time and cause of death. If adjuvant therapy was administered, its type was recorded. Surgical margins were reported as complete or incomplete. As histopathology reports were only reviewed retrospectively, the extent of surgical margins was not quantified. KIT immunohistochemical staining pattern (CD117 A4502 Dako, Denmark) was reported as physiologic (perimembranous) or aberrant (focal or diffuse cytoplasmic). Ki-67 index (MIB-1 M7240; Dako, Denmark) was evaluated as >23 or ≤23 immuno-positive cells/grid area, as previously reported (18).

Available data on follow-up exams, disease-free interval and survival times were collected from the records, by telephone contact with the client and/or the referring veterinarian.

Survival time was defined as time from initial diagnosis (or time of initial presentation at the referral center if not otherwise determinable) to death. Survival was censored for dogs alive at study’s end, dogs lost to follow-up, and dogs with a cause of death unrelated to preputial or scrotal MCT. If cause of death was unknown, death was attributed to the MCT.

Potential prognostic factors evaluated were age, body weight, tumor location (scrotal vs. preputial), neutering status, longest tumor diameter (less than 2 cm vs. greater than or equal to 2 cm), Patnaik (14) and Kiupel (15) histological grade, presence and classification of lymph node metastasis according to the Weishaar Histologic Node (HN) (19) classification system (not evaluated vs. HN0/1/2 vs. HN3), KIT and Ki-67 status, and treatment type (surgery vs. surgery and adjuvant therapy). For the purpose of survival analysis, lymph nodes with “certain metastasis” based on Krick’s cytological criteria (20) were classified under “not evaluated” as long as they did not undergo surgical removal.

### 2.2 Statistical analysis

Data was tested for normality with D’Agostino-Pearson test. The parametric and non-parametric values were compared with the t-test and Mann–Whitney test, respectively. The Fisher’s exact test was used to compare the categorical clinicopathologic features between pMCT and sMCT. Kaplan–Meier method was used to estimate survival time, and the log-rank method was used to compare survival times between groups. When median survival time was not reached, median follow-up time was reported using the reverse Kaplan–Meier method. The influence of potential prognostic variables on overall survival was investigated with univariable and multivariable Cox’s regression analyses. For age and body weight only, generalized additive models were used. Parameters with *p*-values < 0.05 on univariable analysis were tested for independence with multivariable Cox regression analysis.

The statistical analyses were performed with GraphPad Prism version 10 and R software. A *p*-value of < 0.05 was considered statistically significant.

As age was unknown for a single patient, the estimate was interpolated based on the remaining data.

## 3 Results

A medical record search at a single small animal referral hospital identified 169 unique dogs meeting the specified criteria. Ten dogs did not have a confirmed MCT in either of the two locations, 47 had their tumors in the periscrotal or peripreputial location, 15 dogs did not undergo surgical resection of their primary or recurrent MCTs, two MCTs were subcutaneous, one microscopic MCT was identified incidentally only on histopathology after total scrotectomy for castration and was not amenable to grading, and three dogs were lost to follow-up immediately after surgery. One dog presented with microscopic disease following incomplete resection of a grade III scrotal MCT and concurrent urothelial carcinoma of the urinary bladder, and was lost to follow-up after consultation, leaving a total of 91 dogs that met the inclusion criteria.

Clinical data on the 15 dogs that did not undergo surgical resection of their preputial and scrotal MCT are provided in Supplementary materials. All three dogs that were lost to follow-up immediately after surgery had completely resected low-grade MCTs.

Of the 91 dogs that met the inclusion criteria, 35 had preputial and 56 had scrotal MCTs. There were 15 (16.5%) mixed-breed dogs, 13 (14.3%) Retrievers, 11 (12.1%) French Bulldogs, nine (9.9%) Boxers, five (5.5%) Jack Russell Terriers, and 39 (42.6%) dogs belonging to other pure breeds (1–4 dogs per breed). Forty-four dogs with sMCTs were entire and 12 were castrated, while dogs with pMCTs were nearly equally distributed between entire and castrated (18 and 17, respectively). The difference between groups was statistically significant (*p* = 0.011). The median age was 8.0 years, and the mean age was 8.3 years (range: 4–14.25 years). No significant difference in age was found between groups (*p* = 0.540). Median and mean body weights were 27.5 and 26.1 kg, respectively (range 2.6–55 kg; difference between groups *p* = 0.234). Median and mean longest tumor diameters were 1.4 and 1.5 cm, respectively (range 0.5–4 cm; difference between groups *p* = 0.280; data unavailable for 10 dogs). Twenty-seven tumors were at least 2 cm large and 12 tumors (13%) were ulcerated.

Sixty-six (72.5%) dogs presented with a newly diagnosed preputial or scrotal MCT, 19 (20.9%) dogs presented after previous resection and six (6.6%) dogs presented with a recurrent preputial or scrotal MCT. In 51 dogs in which the duration of tumor presence before diagnosis was known, the median and mean durations were 4 and 8.6 months, respectively (range 2 weeks – 50 months; difference between groups *p* = 0.822). In 24 dogs, the MCT was diagnosed within 1 week after discovery by the owners. No information was available for 16 dogs.

### 3.1 Staging

Extirpation and histopathology of the ipsilateral or bilateral superficial inguinal lymph nodes were performed in 50 (54.9%) cases (21/35 or 60.0% of pMCT and 29/56 or 51.8% of sMCT). The lymph nodes were cytologically examined in six additional cases for a total of 56 (61.5%) cases with histopathological or cytological superficial inguinal lymph node evaluation. Twenty-five (27.5%) dogs (11/35 or 31.4% of pMCT and 14/56 or 25% of sMCT; *p* = 0.630) had histologically (14 dogs HN2 and eight dogs HN3) and/or cytologically (3 dogs with “certain metastasis” based on Krick’s criteria (20), all three sMCT) confirmed inguinal lymph node metastases and in one additional case, metastasis was suspected based on marked inguinal lymphadenopathy. Overt (HN3) lymph node metastases occurred more often in pMCT compared to sMCT (5/35 or 14.3% vs. 3/56 or 5.3%, respectively; *p* = 0.252). All but two pMCTs with HN3 metastases were Kiupel low-grade. Two lymph node paraffin blocks (one pMCT and one sMCT) could not be located and the HN classification could not be reviewed; however, based on the original histopathologic report which mentioned a microscopic lymph node metastasis, these cases were classified as HN2 for statistical analysis.

Abdominal staging was performed in 76 (83.5%) dogs. This consisted of an abdominal ultrasound in 75 and a computed tomography scan in a single case. The spleen was aspirated in five and the spleen along with sublumbar lymph nodes were aspirated in one case. In two cases with superficial inguinal lymph node metastases, sublumbar lymph node metastases were also suspected based on imaging. In two other cases (one with suspected sublumbar lymph node involvement), liver and spleen metastasis, respectively, were suspected based on imaging. No visceral metastases were confirmed cytologically.

### 3.2 Histological grades and immunohistochemistry

The majority of MCTs, 45 (49.4%), were Patnaik grade I, 32 (35.2%) were grade II and 14 (15.4%) were grade III. Kiupel grading revealed 76 (83.5%) low-grade and 15 (16.5%) high-grade tumors. Compared to preputial MCTs, scrotal MCTs were more often classified as Patnaik grade III (21.4% vs. 5.7%, *p* = 0.056) and Kiupel high-grade (21.4% vs. 8.6%, *p* = 0.149), respectively. Table 1 shows the distribution of grades between both groups.

**Table 1:** Distribution of tumor grades in 91 dogs with preputial and scrotal cutaneous MCTs.

Tumor grade	Preputial MCT (*n* = 35)	Scrotal MCT (*n* = 56)	Together (*n* = 91)	*p-*value*
Patnaik grade I	22 (62.9%)	23 (41.1%)	45 (49.4%)	/
Patnaik grade II	11 (31.4%)	21 (37.5%)	32 (35.2%)	/
Patnaik grade III	2 (5.7%)	12 (21.4%)	14 (15.4%)	0.056
Kiupel low-grade	32 (91.4%)	44 (78.6%)	76 (83.5%)	/
Kiupel high-grade	3 (8.6%)	12 (21.4%)	15 (16.5%)	0.149

*p-value designates the results of Fisher’s exact test comparing the frequency of tumor grades between both groups.

The median and mean mitotic counts per 10 high-power fields were 1 and 3, respectively (range 1–20; data unavailable for 22 cases).

Immunohistochemistry was performed in 75 (82.4%) cases (28/35 or 80.0% of pMCT and 47/56 or 83.9% of sMCT). The KIT staining pattern was physiological (perimembranous) in 40 (53.3%) and aberrant (cytoplasmic) in 35 (46.7%) tumors. Ki-67 index was ≤23 in 64 (85.3%) and >23 in 11 (14.7%) cases. Numerically, aberrant KIT staining patterns and Ki-67 index >23 were more frequently diagnosed in sMCT than pMCT (*p* = 0.160 and *p* = 0.193, respectively). Table 2 presents the distribution of immunohistochemical results between the two groups.

**Table 2:** Distribution of immunohistochemical results in 91 dogs with preputial and scrotal cutaneous MCTs.

Immunohistochemistry	Preputial MCT (*n* = 28)	Scrotal MCT (*n* = 47)	Together (*n* = 75)	*p-*value*
KIT pattern physiologic	18 (64.3%)	22 (46.8%)	40 (53.3%)	/
KIT pattern aberrant	10 (35.7%)	25 (53.2%)	35 (46.7%)	0.160
Ki-67 index ≤23	26 (92.9%)	38 (80.9%)	64 (85.3%)	/
Ki-67 index >23	2 (7.1%)	9 (19.1%)	11 (14.7%)	0.193

*p-value designates the results of Fisher’s exact test comparing the immunohistochemical results between both groups.

### 3.3 Therapy

According to the inclusion criteria, all dogs were surgically treated. Surgical margins were reported as complete in 80 (87.9%) cases after the initial surgery. In four (4.4%) additional cases, complete margins were attained with a second surgery. Surgical margins were incomplete in three (3.3%) cases. Information on resection margins was unavailable for four (4.4%) cases. Among the three incompletely resected MCTs, two were Kiupel low-grade (Patnaik grade I and II, respectively) and one was Kiupel high-grade / Patnaik grade II.

Twenty-four (26.4%) dogs received some form of adjuvant systemic therapy (detailed in Table 3). The proportion of dogs receiving adjuvant systemic therapy was similar between the groups (pMCT 22.9%, sMCT 26.4%; *p* = 0.630). A single dog with a sMCT received CHOP-based chemotherapy 1.5 years postoperatively due to splenic diffuse large B-cell lymphoma. The remaining 67 (73.6%) dogs were treated with surgery alone.

**Table 3:** Therapeutic interventions in 91 dogs with preputial and scrotal cutaneous MCTs.

Therapy	Preputial MCT (*n* = 35)	Scrotal MCT (*n* = 56)	Together (*n* = 91)
Surgery alone	27 (77.1%)	40 (71.4%)	67 (73.6%)
Surgery + Chemo^a^	/	5 (8.9%)	5 (5.5%)
Surgery + TKI^b^	7 (20.0%)	10 (17.9%)	17 (18.7%)
Surgery + Chemo + TKI	1 (2.9%)	1 (1.8%)	2 (2.2%)

^a^Chemotherapy. ^b^Tyrosine kinase inhibitor.

### 3.4 Outcome and prognostic variables

At data analysis closure, 21 dogs were alive, 16 were lost to follow-up, 14 dogs died due to their MCTs, 27 dogs presumably died due to unrelated causes (no postmortem examinations performed) and in 13 cases the cause of death could not be determined and, therefore, was attributed to the MCT. The overall median survival time was not reached with 1-, 3-, and 5-year survival rates of 85%, 67%, and 60%, respectively (pMCT: 88%, 73%, 66%; sMCT: 83%, 62%, 58%). The survival times between both locations did not differ statistically (*p* = 0.466). The overall median follow-up time was 39 months (range 0.5–110 months; 49 months [range 0.5–110] for pMCT and 34 months [range 1–108] for sMCT).

Factors significantly associated with survival on univariable analysis were age, longest tumor diameter ≥2 cm, Patnaik Grade III, Kiupel high-grade, presence of HN3 lymph node metastases, Ki-67 index >23, aberrant KIT staining pattern, and administration of adjuvant therapy (Table 4). As only three MCTs were incompletely resected, the prognostic impact of incomplete resection was not evaluated. Similarly, different types of adjuvant therapies were not separately evaluated.

**Table 4:** Univariable cox regression analysis of variables potentially associated with increased risk of death in 91 dogs with preputial and scrotal cutaneous MCTs.

Variable	Hazard ratio (95% CI^a^)	*p-*value	MST^b^ in months (95% CI^a^)	Median follow-up in months
Age (years)	1.217 (1.055–1.404)	0.007*	/	/
Body weight	0.994 (0.963–1.026)	0.711	/	/
Location (all)			n.r.^c^	39
Scrotal	1.341 (0.607–2.961)	0.469	n.r.	34
Preputial			n.r.	49
Neuter status				
Neutered	1.544 (0.685–3.480)	0.295	45 (17–∞)	
Entire			n.r.	
Tumor diameter				
Data missing	1.504 (0.418–5.415)	0.533	n.r.	
≥2 cm	3.568 (1.559–8.169)	0.003*	30 (16–∞)	
<2 cm			n.r.	
Kiupel grade				
High-grade	54.830 (11.890–252.800)	<0.001*	9 (5–12)	
Low-grade			n.r.	
Patnaik grade				
I			n.r.	
II	2.731 (1.035–7.206)	0.042	78 (29–∞)	
III	56.111 (13.079–240.726)	<0.001*	10 (5–12)	
KIT staining pattern				
Data missing	3.695 (1.278–10.680)	0.016	45 (12–∞)	
Aberrant	3.844 (1.439–10.260)	0.007*	40 (16–∞)	
Physiologic			n.r.	
Ki-67 index				
Data missing	2.696 (1.097–6.626)	0.031	45 (12–∞)	
>23	8.504 (3.259–22.192)	<0.001*	9.5 (4–∞)	
≤23			n.r.	
LN status				
LN^d^ n.e.^e^	2.441 (0.993–5.998)	0.052	78 (28–∞)	
HN3 LN^b^	5.246 (1.651–16.666)	0.005*	17 (0.5–∞)	
HN0-2 LN			n.r.	
Adjuvant therapy				
Yes	2.540 (1.173–5.498)	0.018*	30 (12–∞)	
No			n.r.	

^a^Confidence interval. ^b^Median survival time. ^c^Not reached. ^d^Lymph node. ^e^Not evaluated histologically. *Significant.

**Figure 1:**
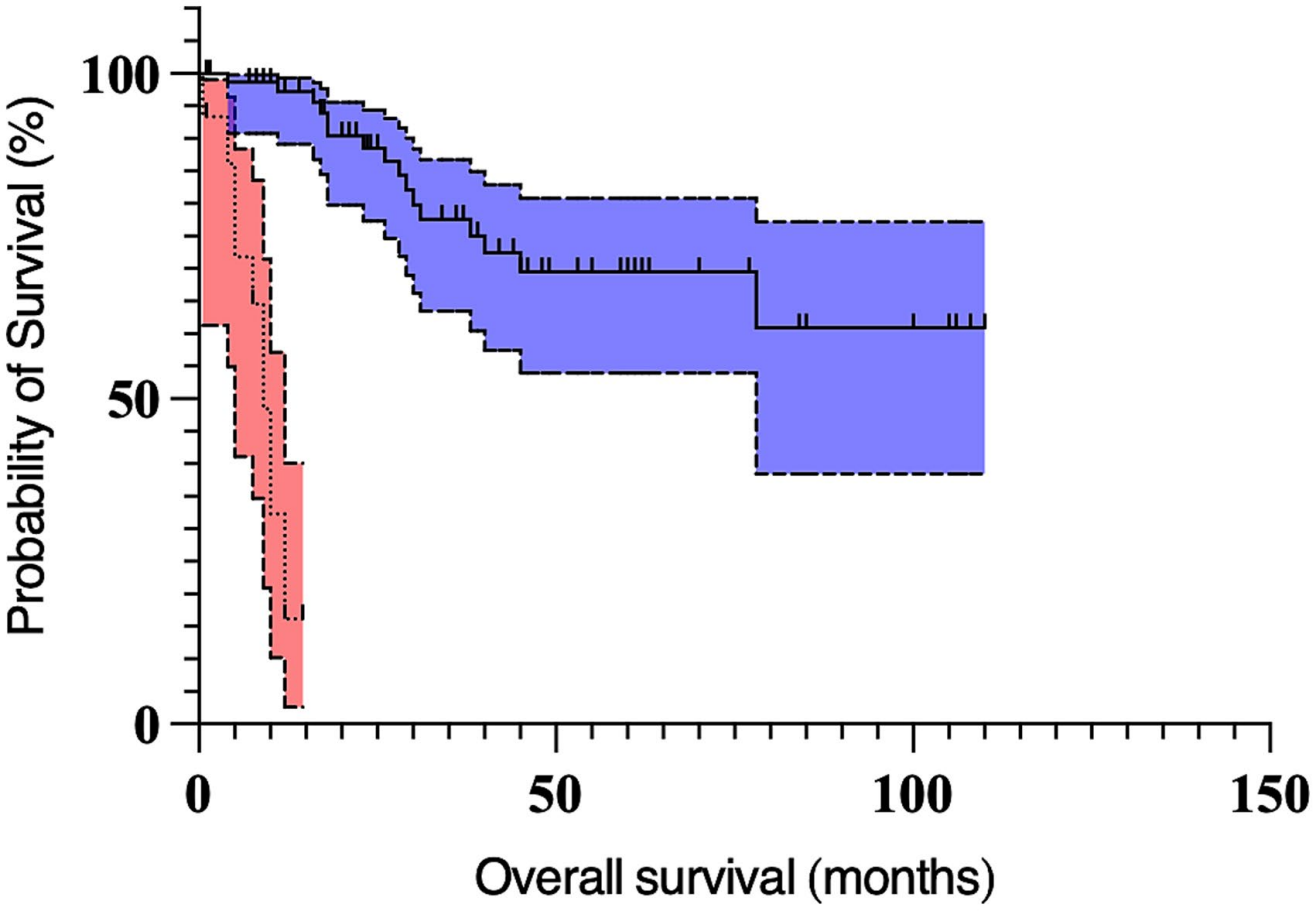
Kaplan Meier curves depicting survival time in dogs with preputial and scrotal cutaneous mast cell tumors with Kiupel high-grade (*n* = 15; dotted line, red 95% confidence interval cloud) and Kiupel low-grade tumors (*n* = 76; solid line, blue 95% confidence interval cloud). The perpendicular ticks represent censored cases. The median survival time for dogs with high-grade tumors (9 months) was significantly shorter (*p* < 0.001) compared to dogs with low-grade tumors (not reached).

**Figure 2:**
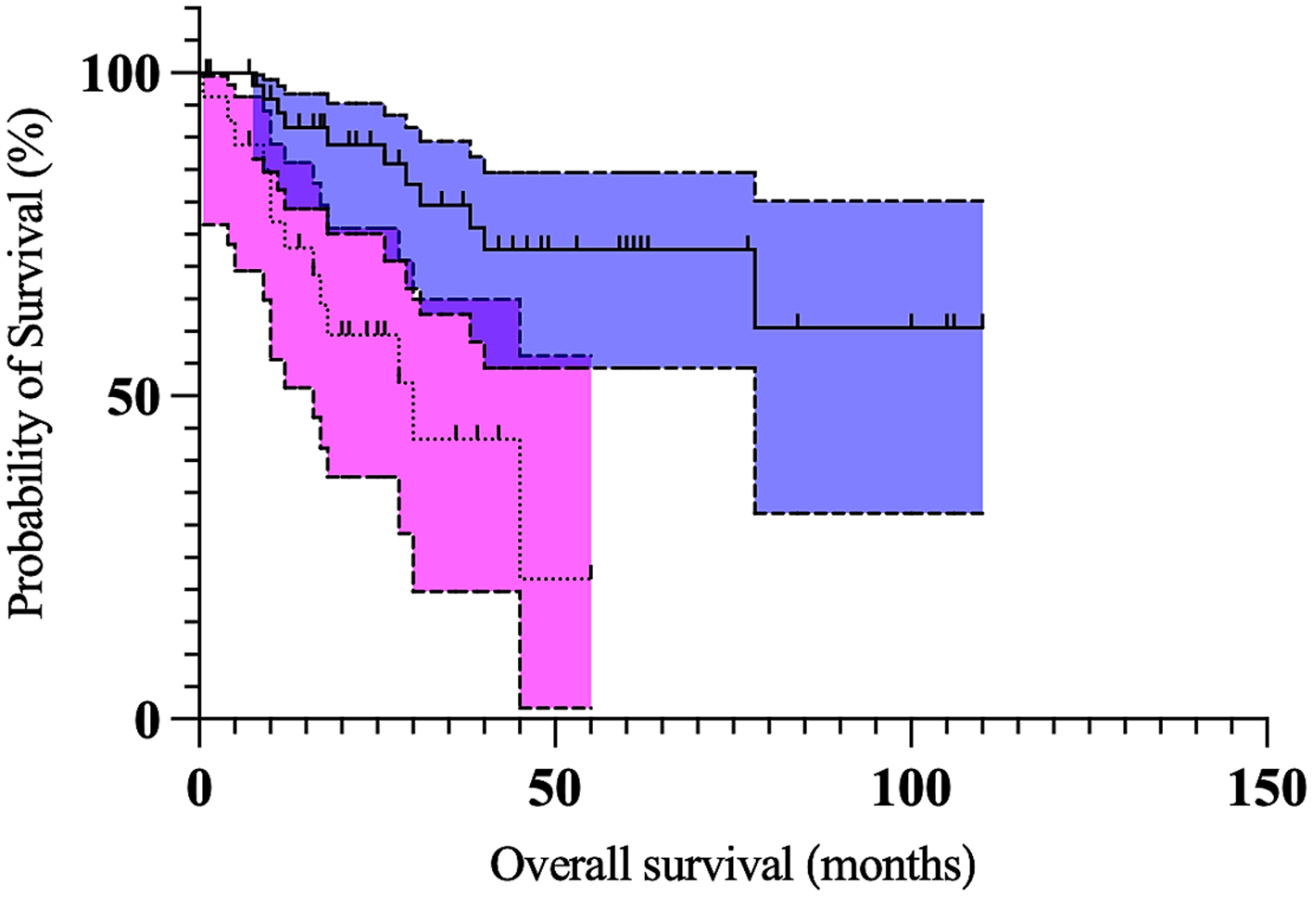
Kaplan Meier curves depicting survival time in dogs with preputial and scrotal cutaneous mast cell tumors with longest tumor diameter ≥2 cm (*n* = 27; dotted line, magenta 95% confidence interval cloud) and longest tumor diameter <2 cm (*n* = 54; solid line, blue 95% confidence interval cloud). The perpendicular ticks represent censored cases. The median survival time for dogs with tumors ≥2 cm (30 months) was significantly shorter (*p =* 0.028) compared to dogs with tumors <2 cm (not reached).

On multivariable analysis, only Kiupel high-grade and the longest tumor diameter ≥2 cm remained significant (Table 5; Figures 1, 2). Due to redundancy and to improve the statistical model, the Patnaik grade was not evaluated in the multivariable analysis.

**Table 5:** Multivariable cox regression analysis of variables associated with increased risk of death in 91 dogs with preputial and scrotal cutaneous MCTs.

Variable	Hazard ratio (95% CI^a^)	*p* value
Age (years)	1.056 (0.881–1.264)	0.558
Tumor diameter ≥2 cm	3.181 (1.131–8.944)	0.028*
Adjuvant therapy	0.641 (0.209–1.967)	0.437
Kiupel high-grade	40.460 (6.549–249.939)	<0.001*
Ki-67 > 23	0.940 (0.223–3.962)	0.932
Aberrant KIT	1.499 (0.425–5.292)	0.530
HN3 LN^b^	2.752 (0.672–11.265)	0.159

Variables with *p* < 0.05 on univariable analysis were included in the model. ^a^Confidence interval. ^b^Lymph node. *Significant.

## 4 Discussion

The prognostic significance of distinct anatomic locations is well established in several canine tumors (21, 22). Compared to most other tumors, a wide array of biologic effects of various anatomic locations have been reported for MCTs (4–10). However, the perception of genital MCTs having a worse prognosis is largely based on anecdotal data (5–7). The aim of our study was to retrospectively evaluate the prognostic implications of preputial and scrotal locations for cutaneous MCTs in by far the largest cohort of dogs thus far.

About 16% of all MCTs were grade III or high-grade (pMCT: 5.7% grade III, 8.6% high-grade; sMCT: 21.4% grade III/high-grade). Even though statistical significance was not reached, scrotal MCTs were histologically more aggressive compared to preputial MCTs with over 20% classified as high-grade (Patnaik grade III and Kiupel high-grade). This is higher than the reported 4.6–12% for MCTs from other anatomic locations, when only considering unbiased populations (5, 23, 24). Hence, a site-specific increased malignancy could be considered for scrotal MCTs. However, higher (>30%) proportions of high-grade/grade III MCTs have been reported for tumors originating from the limbs and the trunk, which have traditionally not been regarded as “high risk” locations (6). In comparison, nearly 50% of pinnal MCTs are reportedly high-grade (10). Unsurprisingly, both Patnaik and Kiupel grades were associated with overall survival. It is possible that a larger number of dogs would have further increased the difference between both sites. Furthermore, to rule out the numerically suggested higher incidence of high-grade MCTs for the scrotal location, a control group of dogs from the same population with MCTs from other cutaneous locations would have to be included.

Inguinal lymph node metastasis was confirmed in just shy of 30% of all dogs in our cohort. Of the 61.5% of dogs with histological and/or cytological evaluation of their inguinal lymph nodes, metastasis [defined as HN2, HN3, or cytological “certain metastasis” based on Krick’s criteria (20)] was identified in 25 of 56 patients (44.6%). The proportion is slightly lower than for some other proposed high risk anatomical locations, e.g., the muzzle (58%) (4), oral and perioral region (72%) (8), oral mucosa (55%) (25), and pinna (56%) (10), which the respective authors have all associated with a higher frequency of lymph node metastasis. However, this proposed higher frequency is not dissimilar compared to more recent studies that systematically evaluated all sentinel lymph nodes in cutaneous and subcutaneous MCTs. Two studies reported a rate of HN2/HN3 lymph node metastasis of 54 and 51% in their cohorts of 66 and 94 dogs with predominantly cutaneous and fewer subcutaneous MCTs, respectively (26, 27). Even though both of these studies included “high risk” locations defined by head, neck, digital, genital and inguinal regions in about a third of all cases, these locations were not found to have a significant impact on the rate of lymph node metastasis. A study evaluating palpably normal regional lymph nodes in 93 dogs with cutaneous MCTs reports an HN2/HN3 lymph node rate of 49.5% (23), which compares well to our data. Since not all inguinal lymph nodes were histologically evaluated in our study, the actual metastatic rate might be different.

Since recent literature suggests that only overt (HN3) nodal metastasis necessitates adjuvant therapy and is, therefore, clinically relevant (27–30), the proportion of HN3 lymph nodes seems most important. Among the previously mentioned studies of high risk locations, only the pinnal MCT study (10) reported the HN lymph node status. Nearly a third of all their dogs had HN3 metastases, which is higher compared to 16% (8/50 with available histology) of dogs in our cohort. Importantly, the reported rate of overt nodal metastasis for cutaneous MCTs of various locations lies between 3 and 21.5% (23, 24, 27). Considering all the above, our data does not show a higher frequency of HN2/HN3 or HN3 lymph node metastasis in preputial and scrotal cutaneous MCTs.

Dogs with HN3 lymph nodes had a median survival time of 17 months. While HN3 lymph node metastasis was associated with a shorter survival on univariable analysis, this was not the case on multivariable analysis. Due to the low proportion of HN3 dogs and few tumor-related deaths the lack of statistical significance could quite possibly be a result of type II error. To find a statistically demonstrable difference, if one exists, an even larger study would be needed. However, favorable prognosis despite HN3 lymph node metastasis has been reported (28, 29) and is, therefore, not unexpected. It is worth noting that 6/8 HN3 dogs received some form of adjuvant systemic therapy. Furthermore, extirpation of these lymph nodes might also have conferred a survival benefit. Consequently, for the purpose of survival analysis, the three dogs with “certain metastasis” (20) based on cytology alone were regarded as “not evaluated” since the reservoir of tumor cells had not been surgically removed.

It is worth pointing out that 6/8 HN3 lymph node metastases were from a low-grade MCT.

One of the main limitations of this study is the inconsistent staging, especially histological and/or cytological evaluation of regional lymph nodes, which might have skewed the evaluation of metastatic spread. As the retrospective data spans over more than two decades, there are notable differences in the diagnostic work-up of earlier patients compared to more recent ones. While in earlier patients lymph node extirpation was mostly performed in cases of lymphadenopathy or clinical suspicion of aggressive disease, surgical removal of ipsilateral or bilateral superficial inguinal lymph nodes - regardless of lymph node size - was more common in more recent patients. This is not unexpected as MCT metastases are not uncommon in palpably normal lymph nodes (4, 23, 27) and routine regional lymph node removal has been suggested as standard of care by some authors (23, 31). The shift toward lymph node extirpation, especially in these “high risk” MCTs in our study, therefore, likely reflects these findings.

As superficial inguinal lymph nodes were histologically and/or cytologically evaluated in less than two-thirds of all dogs, underestimation of microscopic lymphatic spread cannot be ruled out. However, the median survival time of dogs deemed free of lymph node metastasis based solely on palpation and/or ultrasound examination was 78 months. There are several possible explanations for this. Firstly, the dogs with palpably normal lymph nodes could indeed have been free of lymph node metastases. Secondly, the lymph node metastases, if present and missed, were clinically insignificant. In this case, the question remains whether there is any true clinical benefit to prophylactic surgical removal of palpably normal and potentially microscopically metastatic regional lymph nodes in dogs with cutaneous preputial and scrotal MCTs. While the therapeutic role of HN2/HN3 lymph node extirpation has been demonstrated (32), it remains less clear whether extirpation of cytologically negative lymph nodes confers a clinical benefit (31). Human data indicate that the presence of healthy lymph nodes in the drainage area of a tumor plays an important part in maintaining the host’s anti-tumor immunity and that their deliberate removal may actually promote recurrence and/or further metastasis (33, 34). Furthermore, whether HN2 lymph nodes progress to HN3 and, therefore, to a clinically relevant stage after surgical removal of the primary MCT, remains unanswered. Regardless of the explanation for the long follow-up times of dogs without lymph node extirpation in this study, the potential underestimation of microscopic lymph node metastasis seems clinically irrelevant.

Sentinel lymph node mapping was not performed in our cohort and removal of non-sentinel lymph nodes and, hence, missing occult lymph node metastasis remains a possibility. However, based on established canine lymphatic territories and studies on sentinel lymph node mapping in canine MCTs, the superficial inguinal lymph nodes are likely tributary for the majority of canine preputial and scrotal lesions (24, 35).

Besides lymph node extirpation, abdominal staging was not standardized and underestimation of visceral metastasis cannot be excluded, as abdominal imaging without cytology is a poor predictor of hepatosplenic metastasis in canine MCTs (36, 37). However, visceral metastasis has also been shown to be extremely rare in canine cutaneous low risk MCTs and those without sentinel lymph node spread (38, 39). The utility of indiscriminate cytology of abdominal organs in all canine MCTs, therefore, is debatable.

In our cohort, a tumor diameter of 2 cm or larger emerged as an independent negative prognostic factor. Dogs with tumors of at least 2 cm had a 3.2 times increased risk of death. A larger tumor could imply a more aggressive behavior and/or a more rapid growth. It could also suggest a longer-standing disease and, therefore, a potentially increased risk of metastasis. Due to these inherently challenging anatomic locations, large tumors could also render a complete surgical excision impossible. Similar findings have been reported before (26, 28, 32, 39, 40), although different cut-offs have been used. We arbitrarily chose a 2 cm cut-off instead of a more traditionally proposed size of 3 cm, as we believe 3 cm to be too conservative for genital locations. Indeed, only eight tumors were ≥3 cm, possibly pointing toward higher owner vigilance for these locations. An even lower cut-off than 2 cm could have proven prognostic; however, this was not evaluated. Due to the retrospective nature of the study, misclassification of some tumors cannot be ruled out.

The authors agree with previous reports (28) that tumor diameter alone does not justify the addition of adjuvant therapies in cases of complete excision of low-grade tumors, but may warrant more frequent monitoring.

Even though both aberrant KIT staining pattern and Ki-67 index >23 were associated with shorter survival on univariable analysis, these parameters did not retain their significance on multivariable analysis. While both of these markers certainly have clinical utility in select cases, the authors do not believe there is merit in indiscriminate testing in all cutaneous MCTs.

Surprisingly, dogs with scrotal MCTs were more often intact compared to those with preputial MCTs. We believe this to be a result of the fact that in some instances, castration is carried out using total scrotectomy, rendering the development of scrotal MCTs impossible, rather than a true hormonal influence on tumorigenesis in this particular location. However, further studies from ideally other parts of the world are warranted to test this assumption.

Although adjuvant therapy appeared to be a negative prognostic factor in univariable analysis, this was not confirmed in multivariable analysis. Rather than having a true negative effect on overall survival, adjuvant therapy was prescribed in cases of aggressive disease and where a guarded prognosis and a worse outcome were expected.

The overall follow-up time of all dogs in our cohort was over 3 years. Dogs with scrotal MCTs had a shorter follow-up time of 34 months compared to 49 months for dogs with preputial MCTs. The median survival times were not reached and were not statistically different between both groups. However, due to few tumor-related deaths and, consequently, high numbers of censored patients, it cannot be ruled out that an even longer follow-up could have revealed a survival advantage for one location over the other.

Owing primarily to its retrospective nature and inclusion criteria, this study has further limitations besides the ones already discussed. Firstly, we did not include a control group of dogs with MCTs in other cutaneous locations and, therefore, all comparisons are based on historic data. This could make comparisons to dogs with preputial and scrotal MCTs from other parts of the world difficult, as regional differences in disease courses in various populations are possible.

Secondly, only including surgically treated MCTs might have introduced a certain degree of selection bias as dogs with locally extensive or metastasized tumors might have been subjected to medical or palliative treatment only and would, therefore, not be included in the analysis. This could have resulted in missing some aggressive cases and underestimating the prognostic significance of preputial and scrotal locations. However, among the 169 cases initially identified in the database, only four dogs with clinically suspected high-grade tumors due to the presence of metastases and/or extensive local disease or a histologically confirmed high-grade tumor did not undergo surgery. Therefore, the potential selection bias is not likely to have significantly affected the results.

Similarly, as our population only included cases from a referral hospital, we cannot exclude that some advanced cases might not have been referred in the first place and that our population is biased toward those deemed treatable by the referring veterinarians. However, it could also be argued that some clinically less aggressive cases might have been successfully treated by primary care veterinarians. Being the largest referral oncology service in Germany, we believe that our population is a realistic representation of dogs with preputial and scrotal MCTs in Central Europe.

Thirdly, time to tumor progression, tumor-specific survival time and recurrence rate, which are far more objective end-points than overall survival time (41), could not be reliably evaluated from our retrospective data and were omitted. While not ideal, due to a large number of censored patients, overall survival time was the only metric that could be determined with sufficient certainty.

Attributing all deaths of unknown causes to the tumor might be controversial, as it is highly unlikely that all dogs died of the disease. However, we deliberately chose to err on the side of caution as we believe that overestimating the prognostic significance of these anatomic locations is safer for future patients than underestimating it.

Lastly, some possibly important clinical features, e.g., ulceration, could not be reliably determined for all tumors and their effect on survival could not be evaluated.

In conclusion, our data does not provide any evidence of a greater proportion of high-grade tumors or lymph node metastasis and, therefore, an inherently higher biologic aggressiveness of preputial and scrotal MCTs compared to other cutaneous locations. The negative prognostic variables identified (high-grade, larger tumor diameter) are in agreement with the published literature on other cutaneous MCTs. With a median follow-up time of over 3 years and a 5-year survival rate of 60%, dogs in our cohort had an overall favorable prognosis. Despite the challenging locations, surgical resection was curable for a large proportion of dogs with low-grade tumors.

## Data Availability

The raw data supporting the conclusions of this article will be made available by the authors, without undue reservation.

## References

[1] Vail DM. Section D: Myeloma-related disorders In: Withrow SJ Vail DM Page R, editors. Withrow and MacEwen’s Small Animal Clinical Oncology. 6th ed. Amsterdam, Netherlands: Elsevier (2020). 739–52.

[2] London CA Seguin B. Mast cell tumors in the dog. Vet Clin North Am Small Anim Pract. (2003) 33:473–89. doi: 10.1016/S0195-5616(03)00003-2, PMID: 12852232

[3] Blackwood L Murphy S Buracco P de Vos JP de Fornel-Thibaud P Hirschberger J. European consensus document on mast cell tumours in dogs and cats. Vet Comp Oncol. (2012) 10:e1–e29. doi: 10.1111/j.1476-5829.2012.00341.x, PMID: 22882486

[4] Gieger TL Théon AP Werner JA McEntee MC Rassnick KM DeCock HEV. Biologic behavior and prognostic factors for mast cell tumors of the canine muzzle: 24 cases (1990-2001). J Vet Intern Med. (2003) 17:687–92. doi: 10.1111/j.1939-1676.2003.tb02501.x, PMID: 14529136

[5] Sfiligoi G Rassnick KM Scarlett JM Northrup NC Gieger TL. Outcome of dogs with mast cell tumors in the inguinal or perineal region versus other cutaneous locations: 124 cases (1990-2001). J Am Vet Med Assoc. (2005) 226:1368–74. doi: 10.2460/javma.2005.226.1368, PMID: 15844431

[6] Martins AL Carvalho FF Mesquita JR Gärtner F Amorim I. Analysis of risk factors for canine mast cell tumors based on the Kiupel and Patnaik grading system among dogs with skin tumors. Open Vet J. (2021) 11:619–34. doi: 10.5455/OVJ.2021.v11.i4.12, PMID: 35070857 PMC8770182

[7] Śmiech A Ślaska B Łopuszyński W Jasik A Bochyńska D Dąbrowski R. Epidemiological assessment of the risk of canine mast cell tumours based on the Kiupel two-grade malignancy classification. Acta Vet Scand. (2018) 60:70. doi: 10.1186/s13028-018-0424-2, PMID: 30390687 PMC6215678

[8] Hillman LA Garrett LD de Lorimier LP Charney SC Borst LB Fan TM. Biological behavior of oral and perioral mast cell tumors in dogs: 44 cases (1996-2006). J Am Vet Med Assoc. (2010) 237:936–42. doi: 10.2460/javma.237.8.936, PMID: 20946081

[9] Thamm D Turek M Vail D. Outcome and prognostic factors following adjuvant prednisone/vinblastine chemotherapy for high-risk canine mast cell tumour: 61 cases. J Vet Med Sci. (2006) 68:581–7. doi: 10.1292/jvms.68.581, PMID: 16820715

[10] Chalfon C Finotello R Sabattini S Gramer I Morris JS Aralla M. Patterns of nodal metastases, biological behaviour and prognosis of canine mast cell tumours of the pinna: a multi-institutional retrospective study. Vet Comp Oncol. (2023) 21:332–8. doi: 10.1111/vco.12893, PMID: 36907653

[11] Welle M. Development, significance, and heterogeneity of mast cells with particular regard to the mast cell-specific proteases chymase and tryptase. J Leukoc Biol. (1997) 61:233–45. doi: 10.1002/jlb.61.3.233, PMID: 9060446

[12] Welle M Grimm S Suter M Von Tscharner C. Mast cell density and subtypes in the skin of Shar Pei dogs with cutaneous mucinosis. J Veterinary Med Ser A. (1999) 46:309–16. doi: 10.1046/j.1439-0442.1999.00220.x, PMID: 10445005

[13] Cahalane AK Payne S Barber LG Duda LE Henry CJ Mauldin GE. Prognostic factors for survival of dogs with inguinal and perineal mast cell tumors treated surgically with or without adjunctive treatment: 68 cases (1994-2002). J Am Vet Med Assoc. (2004) 225:401. doi: 10.2460/javma.2004.225.401, PMID: 15328716

[14] Patnaik AK Ehler WJ MacEwen EG. Canine cutaneous mast cell tumor: morphologic grading and survival time in 83 dogs. Vet Pathol. (1984) 21:469–74. doi: 10.1177/030098588402100503, PMID: 6435301

[15] Kiupel M Webster JD Bailey KL Best S DeLay J Detrisac CJ. Proposal of a 2-tier histologic grading system for canine cutaneous mast cell tumors to more accurately predict biological behavior. Vet Pathol. (2011) 48:147–55. doi: 10.1177/0300985810386469, PMID: 21062911 PMC8369849

[16] Berlato D Bulman-Fleming J Clifford CA Garrett L Intile J Jones P. Value, limitations, and recommendations for grading of canine cutaneous mast cell tumors: a consensus of the oncology-pathology working group. Vet Pathol. (2021) 58:858–63. doi: 10.1177/03009858211009785, PMID: 33888024

[17] Evans HE de Lahunta A. The urogenital system In: Evans HE, editor. Miller’s anatomy of the dog. 4th ed. Louis, Missouri: Elsevier (2013). 361–405.

[18] Webster JD Yuzbasiyan-Gurkan V Miller RA Kaneene JB Kiupel M. Cellular proliferation in canine cutaneous mast cell tumors: associations with c-KIT and its role in prognostication. Vet Pathol. (2007) 44:298–308. doi: 10.1354/vp.44-3-298, PMID: 17491070

[19] Weishaar KM Thamm DH Worley DR Kamstock DA. Correlation of nodal mast cells with clinical outcome in dogs with mast cell tumour and a proposed classification system for the evaluation of node metastasis. J Comp Pathol. (2014) 151:329–38. doi: 10.1016/j.jcpa.2014.07.004, PMID: 25172053

[20] Krick EL Billings AP Shofer FS Watanabe S Sorenmo KU. Cytological lymph node evaluation in dogs with mast cell tumours: association with grade and survival^*^. Vet Comp Oncol. (2009) 7:130–8. doi: 10.1111/j.1476-5829.2009.00185.x, PMID: 19453367

[21] Polton G Borrego JF Clemente-Vicario F Clifford CA Jagielski D Kessler M. Melanoma of the dog and cat: consensus and guidelines. Front Vet Sci. (2024) 11:1359426. doi: 10.3389/fvets.2024.1359426, PMID: 38645640 PMC11026649

[22] De Nardi AB de Oliveira Massoco Salles Gomes C Fonseca-Alves CE de Paiva FN Linhares LCM Carra GJU. Diagnosis, prognosis, and treatment of canine Hemangiosarcoma: a review based on a consensus organized by the Brazilian Association of Veterinary Oncology, ABROVET. Cancers (Basel). (2023) 15:2025. doi: 10.3390/cancers15072025, PMID: 37046686 PMC10093745

[23] Ferrari R Marconato L Buracco P Boracchi P Giudice C Iussich S. The impact of extirpation of non-palpable/normal-sized regional lymph nodes on staging of canine cutaneous mast cell tumours: a multicentric retrospective study. Vet Comp Oncol. (2018) 16:505–10. doi: 10.1111/vco.12408, PMID: 29893066

[24] Annoni M Borgonovo S Aralla M. Sentinel lymph node mapping in canine mast cell tumours using a preoperative radiographic indirect lymphography: technique description and results in 138 cases. Vet Comp Oncol. (2023) 21:469–81. doi: 10.1111/vco.12906, PMID: 37191042

[25] Elliott JW Cripps P Blackwood L Berlato D Murphy S Grant IA. Canine oral mucosal mast cell tumours. Vet Comp Oncol. (2016) 14:101–11. doi: 10.1111/vco.12071, PMID: 24215587

[26] Ferrari R Boracchi P Chiti LE Manfredi M Giudice C De Zani D. Assessing the risk of nodal metastases in canine integumentary mast cell tumors: is sentinel lymph node biopsy always necessary? Animals (Basel). (2021) 11:2373. doi: 10.3390/ani11082373, PMID: 34438830 PMC8388797

[27] Stefanello D Gariboldi EM Boracchi P Ferrari R Ubiali A De Zani D. Weishaar’s classification system for nodal metastasis in sentinel lymph nodes: clinical outcome in 94 dogs with mast cell tumor. J Vet Intern Med. (2024) 38:1675–85. doi: 10.1111/jvim.16997, PMID: 38426589 PMC11099738

[28] Marconato L Faroni E Battisti E Zaccone R Stefanello D Sabattini S. Incorporation of biologic variables into the staging for canine cutaneous and subcutaneous mast cell Tumours: proposal of the UBo pTNM system. Vet Comp Oncol. (2024) 22:513–22. doi: 10.1111/vco.13000, PMID: 39099458

[29] Guerra D Faroni E Sabattini S Agnoli C Chalfon C Stefanello D. Histologic grade has a higher-weighted value than nodal status as predictor of outcome in dogs with cutaneous mast cell tumours and overtly metastatic sentinel lymph nodes. Vet Comp Oncol. (2022) 20:551–8. doi: 10.1111/vco.12806, PMID: 35195937

[30] Marconato L Stefanello D Kiupel M Finotello R Polton G Massari F. Adjuvant medical therapy provides no therapeutic benefit in the treatment of dogs with low-grade mast cell tumours and early nodal metastasis undergoing surgery. Vet Comp Oncol. (2020) 18:409–15. doi: 10.1111/vco.12566, PMID: 31930651

[31] Sabattini S Kiupel M Finotello R Stefanello D Faroni E Bertazzolo W. A retrospective study on prophylactic regional lymphadenectomy versus nodal observation only in the management of dogs with stage I, completely resected, low-grade cutaneous mast cell tumors. BMC Vet Res. (2021) 17:331. doi: 10.1186/s12917-021-03043-0, PMID: 34649575 PMC8518262

[32] Marconato L Polton G Stefanello D Morello E Ferrari R Henriques J. Therapeutic impact of regional lymphadenectomy in canine stage II cutaneous mast cell tumours. Vet Comp Oncol. (2018) 16:580–9. doi: 10.1111/vco.12425, PMID: 30047226

[33] Darragh LB Gadwa J Pham TT Van Court B Neupert B Olimpo NA. Elective nodal irradiation mitigates local and systemic immunity generated by combination radiation and immunotherapy in head and neck tumors. Nat Commun. (2022) 13:7015. doi: 10.1038/s41467-022-34676-w, PMID: 36385142 PMC9668826

[34] Inamori K Togashi Y Fukuoka S Akagi K Ogasawara K Irie T. Importance of lymph node immune responses in MSI-H/dMMR colorectal cancer. JCI Insight. (2021) 6:137365. doi: 10.1172/jci.insight.137365, PMID: 33755600 PMC8262295

[35] Suami H Yamashita S Soto-Miranda MA Chang DW. Lymphatic territories (Lymphosomes) in a canine: an animal model for investigation of postoperative lymphatic alterations. PLoS One. (2013) 8:e69222. doi: 10.1371/journal.pone.0069222, PMID: 23894435 PMC3722290

[36] Hughes JR Szladovits B Drees R. Abdominal CT evaluation of the liver and spleen for staging mast cell tumors in dogs yields nonspecific results. Vet Radiol Ultrasound. (2019) 60:306–15. doi: 10.1111/vru.12717, PMID: 30786323

[37] Pecceu E Serra Varela JC Handel I Piccinelli C Milne E Lawrence J. Ultrasound is a poor predictor of early or overt liver or spleen metastasis in dogs with high-risk mast cell tumours. Vet Comp Oncol. (2020) 18:389–401. doi: 10.1111/vco.12563, PMID: 31863546

[38] Rinaldi V Crisi PE Vignoli M Pierini A Terragni R Cabibbo E. The role of fine needle aspiration of liver and spleen in the staging of low-grade canine cutaneous mast cell tumor. Vet Sci. (2022) 9:473. doi: 10.3390/vetsci9090473, PMID: 36136689 PMC9506313

[39] Fejös C Troedson K Ignatenko N Zablotski Y Hirschberger J. Extensive staging has no prognostic value in dogs with low-risk mast cell tumours. Vet Comp Oncol. (2022) 20:265–75. doi: 10.1111/vco.12773, PMID: 34564910

[40] Moore AS Frimberger AE Taylor D Sullivan N. Retrospective outcome evaluation for dogs with surgically excised, solitary Kiupel high-grade, cutaneous mast cell tumours. Vet Comp Oncol. (2020) 18:402–8. doi: 10.1111/vco.12565, PMID: 31916687

[41] Delgado A Guddati AK. Clinical endpoints in oncology - a primer. Am J Cancer Res. (2021) 11:1121–31. PMID: 33948349 PMC8085844

