# Supplementary material for: Preputial and scrotal cutaneous mast cell tumors in dogs show no evidence of inherently higher biologic malignancy
Source: Front Vet Sci. 2025 Oct 29;12:1672099. doi: 10.3389/fvets.2025.1672099 (PMC12609186; doi:10.3389/fvets.2025.1672099)
Supplement: Supplementary file 2 [file Table_1.DOCX]

Supplementary Material

# Supplementary Data

Among the 15 dogs excluded because they did not undergo surgical resection of their macroscopic MCTs, two presented with recurrence after incomplete resection by the primary care veterinarian (one scrotal low and one scrotal high-grade MCT) and were lost to follow-up after consultation, one dog had a biopsy performed (grade I/low-grade) and was treated systemically with masitinib and lost to follow-up after 44 months, one dog was presented to emergency service due to severe swelling of its preputial MCT five days after biopsy (grade II/low-grade) and was euthanized at owner’s request due to micturition issues. Five dogs had cytologically well-differentiated MCTs (two with pMCTs lost to follow-up after consultation, one with sMCT euthanized after 46 months due to chronic kidney disease and one with pMCT lost to follow-up after 52 months [both without any MCT-related therapy], and one with sMCT euthanized after 4 months due to local progression and systemic signs of weakness). One dog’s scrotal MCT was cytologically moderately pleomorphic and moderately granulated and remained stable for 14 months, after which the dog was lost to follow-up. Another dog’s sMCT was deemed only marginally resectable and was treated with masitinib. The dog was lost to follow-up after 8 months with signs of local progression. Three dogs presented with clinically and/or cytologically suspected high-grade MCTs with extensive local disease and regional lymph node metastasis. In all three cases, systemic therapy was recommended, but the dogs were lost to follow-up after consultation.
